# A unified censored normal regression model for qPCR differential gene expression analysis

**DOI:** 10.1371/journal.pone.0182832

**Published:** 2017-08-17

**Authors:** Peter Pipelers, Lieven Clement, Matthijs Vynck, Jan Hellemans, Jo Vandesompele, Olivier Thas

**Affiliations:** 1 Department of Mathematical Modelling, Statistics and Bioinformatics, Ghent University, Ghent, Belgium; 2 Department of Applied Mathematics, Computer Science and Statistics, Ghent University, Ghent, Belgium; 3 Bioinformatics Institute Ghent from Nucleotides to Networks (BIG N2N), Ghent University, Ghent, Belgium; 4 Center for Medical Genetics, Ghent University, Ghent, Belgium; 5 Biogazelle, Zwijnaarde, Belgium; 6 National Institute for Applied Statistics Research Australia (NIASRA), School of Mathematics and Applied Statistics, University of Wollongong, Wollongong, NSW 2522, Australia; University of Florida, UNITED STATES

## Abstract

Reverse transcription quantitative polymerase chain reaction (RT-qPCR) is considered as the gold standard for accurate, sensitive, and fast measurement of gene expression. Prior to downstream statistical analysis, RT-qPCR fluorescence amplification curves are summarized into one single value, the quantification cycle (*Cq*). When RT-qPCR does not reach the limit of detection, the *Cq* is labeled as “undetermined”. Current state of the art qPCR data analysis pipelines acknowledge the importance of normalization for removing non-biological sample to sample variation in the *Cq* values. However, their strategies for handling undetermined *Cq* values are very ad hoc. We show that popular methods for handling undetermined values can have a severe impact on the downstream differential expression analysis. They introduce a considerable bias and suffer from a lower precision. We propose a novel method that unites preprocessing and differential expression analysis in a single statistical model that provides a rigorous way for handling undetermined *Cq* values. We compare our method with existing approaches in a simulation study and on published microRNA and mRNA gene expression datasets. We show that our method outperforms traditional RT-qPCR differential expression analysis pipelines in the presence of undetermined values, both in terms of accuracy and precision.

## 1 Introduction

High-throughput reverse transcription quantitative polymerase chain reaction (RT-qPCR) is a popular technology for gene expression profiling. An important advantage of qPCR is the speed, specificity and sensitivity of the qPCR assays. qPCR is often referred to as the gold standard for gene expression profiling [e.g. [1]]. Therefore, it is commonly used within the context of diagnostic and prognostic testing as well as for biological validation of biomarkers discovered in large screening experiments with microarray or next generation sequencing technologies. RT-qPCR is a cyclic process in which targeted molecules are amplified and simultaneously quantified by measuring a fluorescence intensity. The raw RT-qPCR profiles are typically summarized into a single value, the quantification cycle *Cq*. Common procedures for calculating *Cq*-values are based on the number of cycles needed to cross a certain threshold, or on second derivatives of the amplification curve [e.g. [2]]. If a target is not expressed or the amplification step fails, the threshold is not reached after the maximum number of cycles (limit of detection or LOD) and the *Cq* is undetermined. A typical qPCR dataset thus consists of both expressed and undetermined *Cq*-values (UV). To our knowledge, the existing methods for handling UV in qPCR experiments are very ad hoc. Popular approaches either remove UV or perform imputation. The former approach ignores data that is informative whereas the latter results in artifacts. Although the truncation at the LOD has implications for all types of subsequent data analysis (e.g. cluster analysis, absolute quantification, …), our method is specifically developed for differential expression analysis.

Similar to other gene expression workflows, differential expression analysis with qPCR involves a) preprocessing, b) statistical analysis and c) correction for multiple testing. The existing qPCR analysis pipelines are sequential: first, technical sample variation is reduced in a separate normalization step and subsequently multiple hypothesis tests are conducted. Although the effect of the pre-processing can be quite substantial, it is typically ignored in the subsequent analysis steps. This can imply an incorrect control of the significance level *α*, which can lead to an increased false positive rate or a reduced power. Within a microarray context, it has been shown that error propagation can improve the accuracy of the differential expression analysis substantially. But, even when error propagation is provided, the analysis can still be suboptimal: each step in a modular approach is optimized individually without taking the previous and the future analysis steps into account [3].

We present a unified censored normal regression (UCNR) model for differential expression analysis of RT-qPCR data. In contrast to a sequential approach, a “unified” method simultaneously performs normalization and statistical testing, while correctly accounting for UV. In the presence of UV, our method outperforms state of the art RT-qPCR data analysis pipelines in terms of accuracy and precision. Our hypothesis tests are more robust to UV and the method increases the stability of the estimated normalization factors. The paper is organised as follows: we introduce the unified censored normal regression model and elaborate on the interpretation of the parameters for differential microRNA/gene expression. Next, we compare the robustness of hypothesis testing and normalization of our model-based approach to popular methods for analysing RT-qPCR data. Finally, we illustrate the method on a microRNA gene expression study in neuroblastoma to find differentially expressed genes between *MYCN* amplified and *MYCN* single copy tumor samples [4], and on a mRNA gene expression study in neuroblastoma to detect differentially expressed prognostic genes between patients with higher risk of death from disease or higher risk of relapse or progression and patients with low risk [5].

## 2 Materials and methods

MicroRNA gene expression data in neuroblastoma (NB) with 430 profiled microRNAs and 18 small RNA controls, as described in [4], is used to set up a simulation study and analysed as a case study for differential expression analysis between two tumor groups in 61 samples, with 22 *MYCN* amplified (MNA) and 39 *MYCN* single copy (MNSC) tumor samples (61 samples × 448 microRNAs = 27328 observations). A second case study involves a cohort of 343 neuroblastoma patients from a study of the International Society of Pediatric Oncology, European Neuroblastoma Group (SIOPEN) with 58 prognostic genes, that serves as a multigene-expression signature for patients with neuroblastoma [5], and 5 reference genes. Finally, we consider an independent cohort of 236 patients from the Children’s Oncology Group (COG) that was used as validation cohort for testing the multigene-expression signature [5]. The raw miRNA expression data, experimental annotation and sample annotation are available at https://github.com/CenterForStatistics-UGent/UCNR.

## 3 Results

### 3.1 Unified censored normal regression model

From a statistical point of view, UV can be considered as right censored, i.e. the data is incompletely observed, but UV are known to correspond with a *Cq* of at least LOD cycles (e.g. LOD = 40); we thus observe an undetermined value as the LOD. Suppose that C* represents the hypothetical *Cq* value that would have been observed if there was no LOD. In statistical terms the partly unobservable C* is referred to as a latent process. Our approach consists in modelling this latent process and the parameters of this model are expected to give an unbiased assessment of differential expression.

Formally, if *C* refers to the observed *Cq* and C* to the latent *Cq* process, then they are related through

$$\begin{array}{c} C=\min(C^{*},LOD). \end{array}$$ (1)

Consider a study with *J* samples and *I* targets. The samples are divided into *K* groups and the objective of the study is testing for differential expression between the groups. The censored regression model is a hierarchical statistical model. On the top layer, Eq (1) relates the observed *C* to the latent process C*. The latter is further modeled as a classical linear model. In particular,

$$\begin{array}{c} C_{ijk}^{*}=\mu +\alpha_{i}+\beta_{j}+{\left(\alpha \gamma \right)}_{ik}+\varepsilon_{ij}, \end{array}$$ (2)

where

- *μ*: intercept
- *α*_*i*_: effect of target *i*, with *i* = 1, …, *I*
- *β*_*j*_: effect of sample *j*, with *j* = 1, …, *J*
- *αγ*_*ik*_: interaction effect between target *i* and group *k*, with *k* = 1, …, *K*
- $\varepsilon_{ij}∼N\left(0,\sigma_{i}^{2}\right)$: error term reflecting the random noise.

While the sample effect *β*_*j*_ is included for preprocessing purposes, the interaction effect between target *i* and group *k* is a measure for differential expression of target *i* between a reference group and the group *k* of interest (see S1 Appendix). The parameter *β*_*j*_ represents a normalization factor that is similar to the modified global mean [6], which is an improvement of the global mean strategy [4]. The modified global mean procedure (MOD) consists of (a) centering the responses within each target for attributing an equal weight to each target in the subsequent normalisation; (b) centering the modified responses of (a) within each sample around the mean of all expressed targets (or around the mean of the targets expressed in all samples). MOD results in adequate removal of technical variability, as evidenced by more pronounced and balanced differential expression [6].

The model defined by Eqs (1) and (2) is referred to as the UCNR model, which is a variation of the Tobit model [7]. Parameters can be estimated by means of maximum likelihood [8]. The maximum likelihood estimators are consistent and asymptotically normal. The UCNR can be used for testing differential expression of targets and also for the estimation of differential expression in terms of the log_2_ fold change, while simultaneously normalizing the data. For target *i*, this log_2_ fold change is denoted by *δ*_*i*_, which is a contrast of the interaction effect parameters (see S1 Appendix). Within our framework, generalised Wald tests for *H*_0_: *δ*_*i*_ = 0 can be used for assessing differentially expressed targets. In the absence of UV this procedure is very similar to a sequential analysis that exists in MOD normalized data followed by a *t*-test (see S1 Appendix).

The estimator of *δ*_*i*_ takes the UV correctly into account. Treating UV as censored observations has the advantage that we continue working with all raw observed data and no ad hoc data manipulations are required. We illustrate the robustness of the estimator in the presence of UV by means of a simulation study with real data characteristics and compare it with classical sequential analyses. To make both methods comparable, we consider classical analyses using multiple *t*-tests, i.e. a single *t*-test for testing for differential expression for each target.

In the RT-qPCR literature, there is no consensus on preprocessing UV. Imputation of the undetermined *Cq* values by the LOD is suggested [9], while other approaches rely on a regression to the mean. In a simulation study we consider three common strategies for handling UV. The first strategy imputes the undetermined value by the LOD and normalizes the imputed values by subtracting the modified global mean or the mean of the selected reference genes (LOD). A second strategy imputes the UV by the maximum normalized value of each individual target and adds 1 so as to preserve the undetermined realization (MNV+1). The third strategy is a *k*-nearest neighbor algorithm that determines the *k* nearest neighbors for a gene using a Euclidian metric and imputes the UV by the average of the normalized expressed values of its neighbors (KNN). The latter strategy is provided within the SAM algorithm [10] for imputing missing values.

### 3.2 Robustness of the differential expression estimator in the presence of undetermined values

We conducted a simulation study based on the microRNA gene expression NB data [4]. From the total of 448 microRNAs profiled in the NB set, we discarded the microRNAs with at least 1 UV regardless of the group (MNA or MNSC) and only considered the remaining set of 201 microRNAs. For each individual microRNA, we shifted one group to equalize the mean normalized *Cq*-values (MOD) in the two groups. For computational reasons and optimal graphical display, we considered a random subset of 50 microRNAs from which we altered 20 microRNAs by adding a *δ*_*i*_ = 2 or *δ*_*i*_ = −2 differential expression to the *Cq* values in one group. We divided the number of up- and downregulated microRNAs equally over the study. The difference *δ*_*i*_ is thus interpreted as a linear fold change of 4. The remaining 30 microRNAs are not differentially expressed (*δ*_*i*_ = 0). The *δ* parameters in this uncensored data set (*δ*_*i*_ = 0 or *δ*_*i*_ = ±2) are considered as the true parameter values. Hence, a total of 61 samples × 50 microRNAs = 3050 observations are involved.

**Fig 1:**
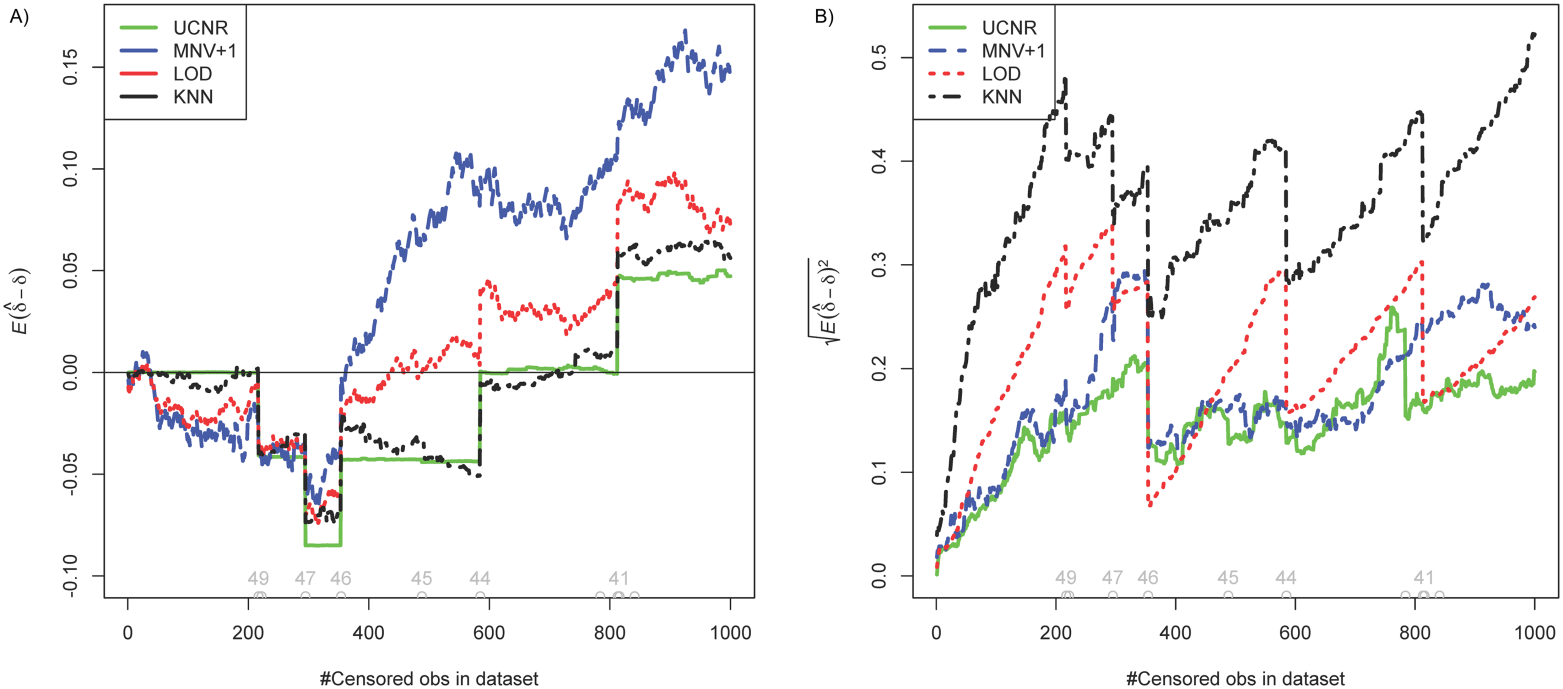
(A) Bias and (B) RMSE of the differential expression estimates of 50 microRNAs, as a function of the number of censored *Cq* values. At the bottom the grey circles indicate the removal of a complete miRNA (as a consequence of censoring). The numbers on top of some of the grey circles represent the number of remaining miRNA in the study. Estimators are obtained by UCNR (green solid line), multiple *t*-tests with MOD normalization and LOD imputation of the UV (red dashed line), multiple *t*-tests with MOD normalization and MNV+1 imputation (blue dotted line) and multiple *t*-tests with MOD normalization and KNN imputation (black dotted-dashed line). A bias closer to zero suggest more accurate estimates. A small RMSE indicate a high precision of the estimator. The sharp jumps in the curves happen when a complete miRNA gets censored, which heavily affects the normalisation constants.

We evaluated the impact of UV for the different methods in an iterative procedure. First the LOD is set to the largest *Cq* value in the dataset. At each step *s* = 1, …, 1000 of the procedure, we censored the maximum uncensored observation in the dataset, resulting in a stepwise decrease of the LOD, and we test for differential expression of the microRNAs using the UCNR and the classical analyses with the three imputation strategies (LOD, MNV+1 and KNN with *k* = 10). At the end of the procedure, about a third of the data is censored. We evaluate the estimates ${\hat{\delta }}_{i}$ in Fig 1. The study illustrates the robustness of the estimator of differential expression in the presence of UV (Fig 1). For iteration *s*, each analysis provides a set of estimates ${\hat{\delta }}_{i}$ of the parameters *δ*_*i*_. Fig 1A tracks the mean difference between *δ*_*i*_ and ${\hat{\delta }}_{i}$, which is an estimate of the bias, while Fig 1B tracks the square root of the mean squared error (RMSE). Both statistics assess the robustness of the estimators in the presence of UV: the smaller the bias and the RMSE the better the estimator. The graphs suggest that the bias of the estimator obtained by UCNR is minimal and approximately piecewise constant in this study. The estimators provided by the sequential analyses fluctuate heavily due to the ad hoc imputations. Fig 1B shows a smaller RMSE for our new estimator in comparison with the classical analyses. One may argue that in a small interval (between about 300 to 600 steps) UCNR seems to have a slightly larger bias as compared to the other methods, but in this interval the UCNR method has a very good precision (Fig 1). Both graphs demonstrate the robustness of the new estimator (UCNR) of differential expression in the presence of UV, which improves upon the traditional approaches with respect to both accuracy and precision.

S1 and S2 Figs show results for the bias and the RMSE in a similar simulation study, but starting from 100 and 200 completely observed microRNAs (i.e. no UV at the start of the simulations). The conclusions are the same as for the 50 microRNA setting, demonstrating the scalability of the method.

### 3.3 Robustness of the hypothesis tests in the presence of undetermined values

**Fig 2:**
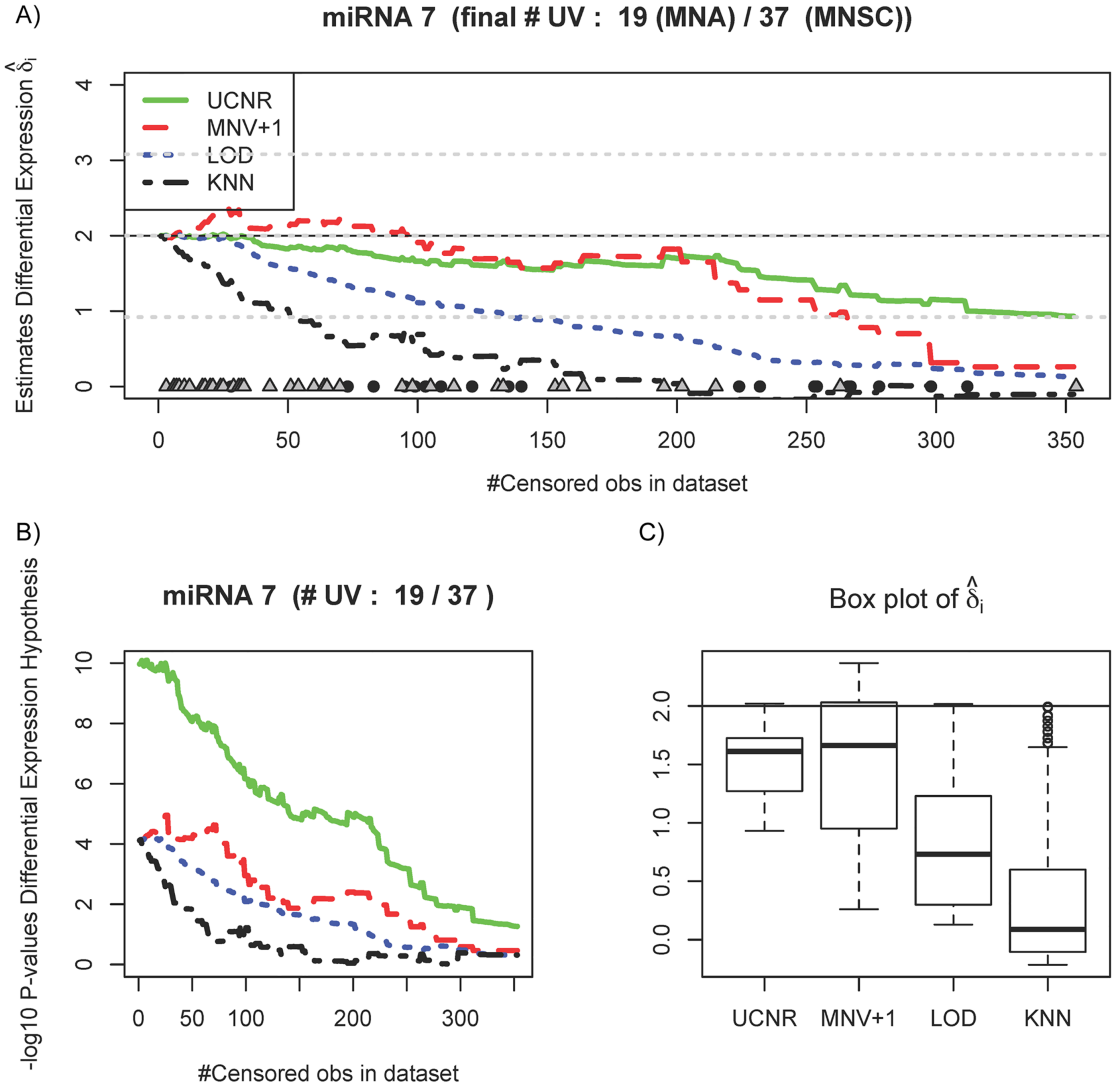
A differentially expressed microRNA (true *δ*_*i*_ = 2) tracked during the simulation study. (A) Estimates of differential expression by UCNR (green solid line), multiple *t*-tests with MOD normalization and LOD imputation (red dashed line), MNV+1 imputation (blue dotted line) and KNN imputation (black dotted-dashed line). Censoring an observation at some point for this particular microRNA is marked by a black circle (MNA group) or a grey square (MNSC group) on the horizontal axis. (B) Plot of − log_10_ *p*-values for the hypothesis test (*H*_0_: *δ*_*i*_ = 0; *H*_1_: *δ*_*i*_ ≠ 0). (C) Box plot of differential expression estimates.

**Fig 3:**
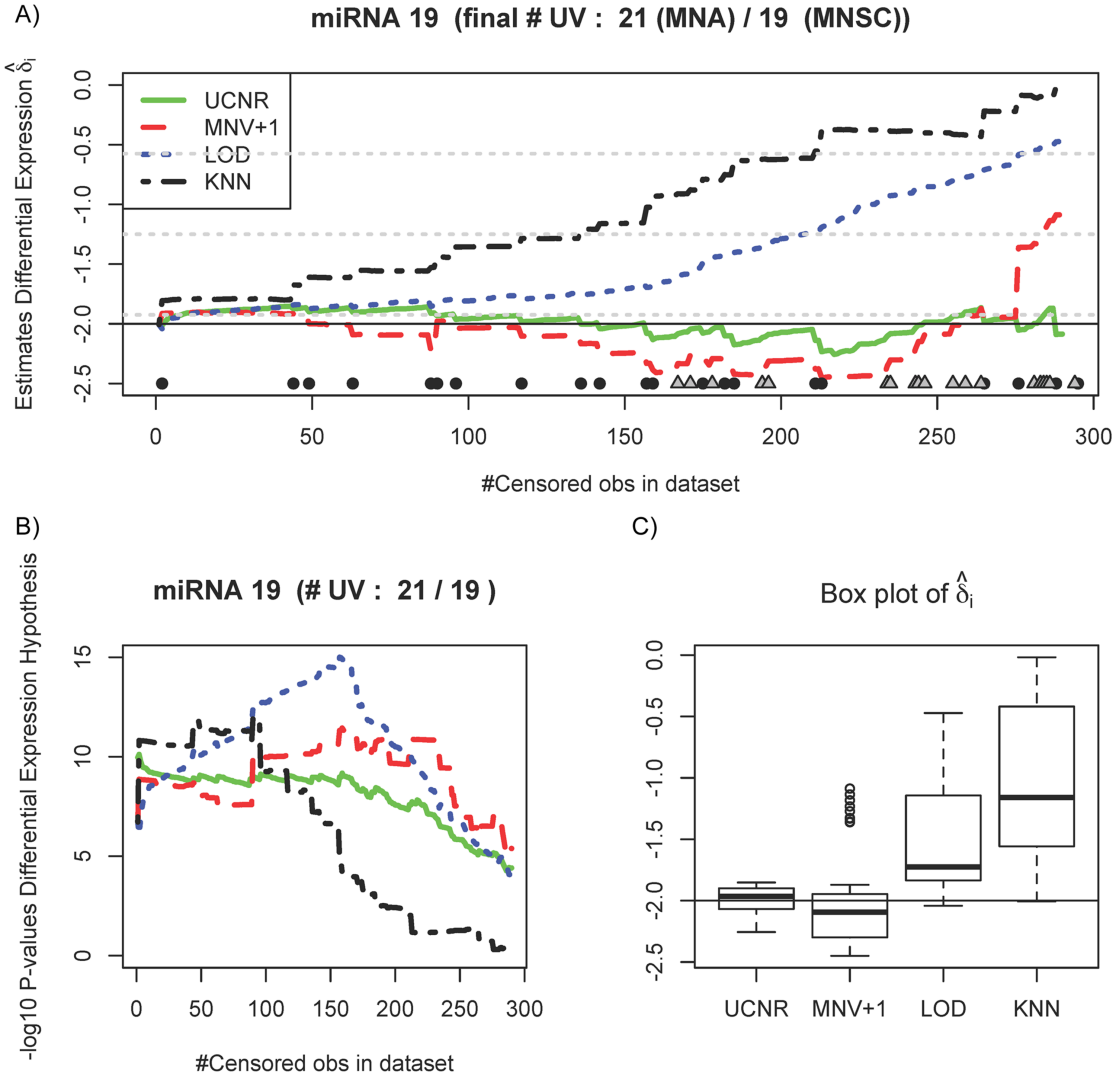
A differentially expressed microRNA (true *δ*_*i*_ = −2) tracked during the simulation study. (A) Estimates of differential expression by UCNR (green solid line), multiple *t*-tests with MOD normalization and LOD imputation (red dashed line), MNV+1 imputation (blue dotted line) and KNN imputation (black dotted-dashed line). Censoring an observation at some point for this particular microRNA is marked by a black circle (MNA group) or a grey square (MNSC group) on the horizontal axis. (B) Plot of − log_10_ *p*-values for the hypothesis test (*H*_0_: *δ*_*i*_ = 0; *H*_1_: *δ*_*i*_ ≠ 0). (C) Box plot of differential expression estimates.

Using the same data and simulation setup as in the previous paragraph, Figs 2 and 3 illustrate the behaviour of individual hypothesis tests for differential expression (*H*_0_: *δ*_*i*_ = 0; *H*_1_: *δ*_*i*_ ≠ 0). The figures track the differential expression estimates and *p*-values for two representative targets for which *δ*_*i*_ = ±2, for both the classical sequential approaches with multiple *t*-tests and the UCNR. As before, the estimator from the UCNR is more robust in the presence of UV, and improves upon the sequential approaches in terms of accuracy and precision. This is also reflected in the UCNR *p*-values (Fig 2(b)), that do not vary as much as with the sequential approaches and remain significant even when a large fraction of the *C*_*q*_ values is set to UV. The uncertainty of the estimates from the four approaches is shown in a box plot (Fig 2(c)). After the introduction of approximately 350 UV the microRNA was removed from the study due to the large amount of censoring (19 observations in the MNA group and 37 in the MNSC group), which is common practice when too many datapoints are missing (typically 80% or more).

In the presence of UV, the estimates of differential expression with the sequential approaches differ due to the different imputation strategies. Imputation with MNV+1 treats the UV as ties, which explains the better performance of this estimator and an increase of the *p*-values only at the end. The estimates of the four analyses tend towards zero as censoring increases, but the bias is clearly larger for the sequential analyses. The *p*-values obtained from classical analyses inflate heavily at low levels of censoring, while the *p*-values of the UCNR remain stable over a larger range of censoring. Correctly rejecting the null hypothesis is more often guaranteed with the UCNR. Intuitively, the proportion of censored observations that a statistical test can handle before a type II error (falsely accept *H*_0_) is committed, indicates the robustness of the hypothesis test. For the sequential analyses, these proportions are 0.69 (LOD), 0.79 (MNV+1) and 0.34 (KNN), while for UCNR this proportion is equal to 0.90. Larger values indicate more robustness. Fig 3 shows the results for a microRNA (true *δ*_*i*_ = −2) that is heavily censored in the MNA-group and is removed from the study after approximately 300 steps. The *p*-values (Fig 3(b)) remain constant and significant (5% significance level) with increasing censoring for both UCNR and the sequential analyses, except for KNN for which the *p*-values diverge. Note that the bias of the estimator of *δ*_*i*_ remains small for UCNR, while the sequential analyses provide estimates that again tend towards zero as censoring increases (Fig 3(a)), because they rely on the observations of both groups for the ad hoc imputation of UV.

With a sequential method it is also possible to apply a Wilcoxon rank sum test after normalisation. In S3 Fig results are presented from the same simulation study, but with the *t*-test replaced by the Wilcoxon rank sum test after MNV+1, LOD or KNN normalisation. Again the *p*-values of the UCNR method remain more stable with increasing censoring.

### 3.4 Latent mean normalization

**Fig 4:**
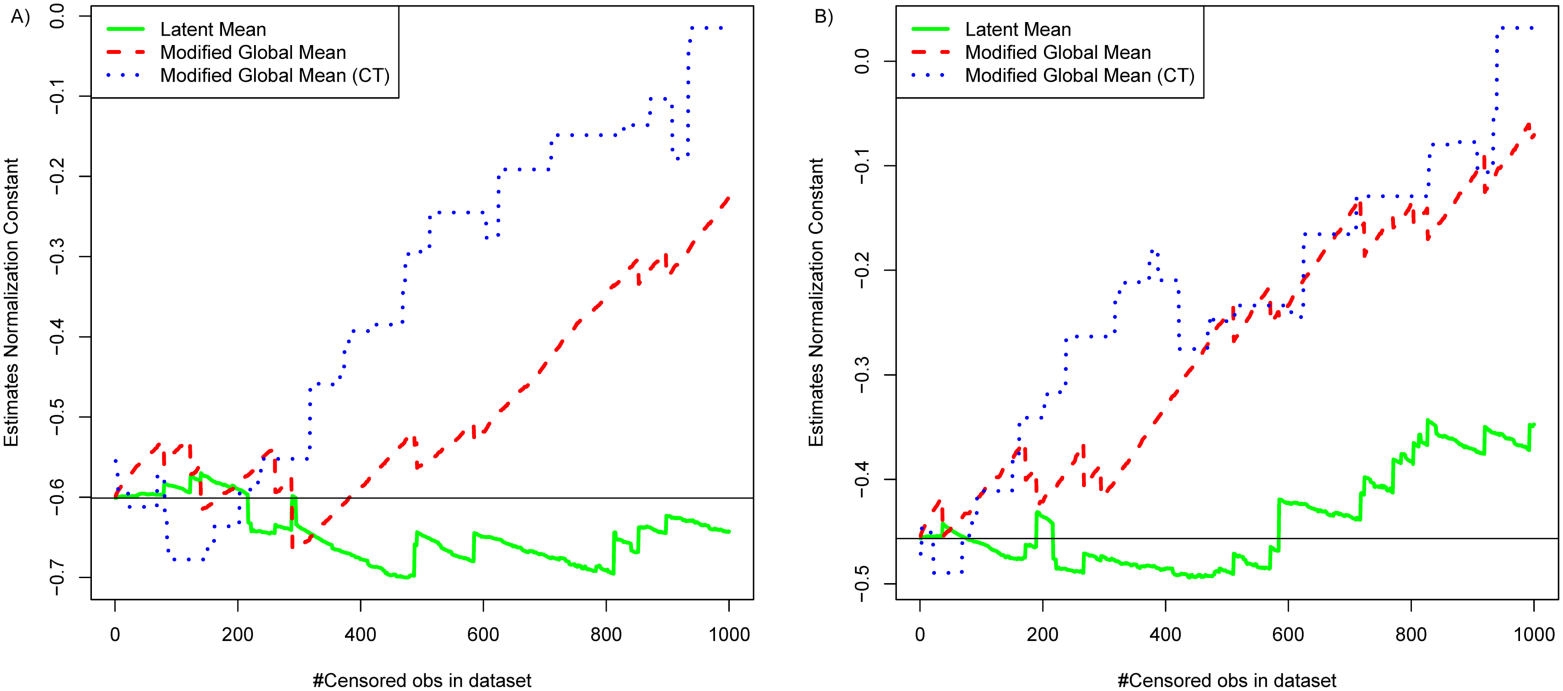
Estimates of the normalization factor of two representative samples ((A) sample 2, (B) sample 3) in the simulation study. Estimates are obtained by LMN (green solid line), MOD normalization (red dashed line) and MOD normalization on common targets (blue dotted line). The true normalization factor is represented by the horizontal line.

Centering the response around the global mean of the expressed microRNAs adequately removes technical variation and reduces the number of false negatives [4]. This normalization procedure is further improved by first centering the targets and thus attributing equal weight to the individual targets [6]. Our model extends this approach by including the UV. In particular, the estimator of the *β*_*j*_ parameter in the UCNR model (2) has the interpretation of a normalisation factor for sample *j*. The robustness of this estimator (${\hat{\beta }}_{j}$) is illustrated in Fig 4 which tracks the estimates of the true normalization factor for two representative samples in the simulation study obtained by UCNR, MOD normalization and MOD normalization on common targets. The latter computes the normalization factor using only the targets that are expressed in all samples. Since the censored observations are not considered, both MOD normalization estimates rapidly diverge from the true normalization factor (Fig 4), which explains the peaks and the crossing curves in Fig 1. The UCNR method requires no imputations and takes the uncertainty of the estimates into account. We refer to this normalization procedure as *latent mean normalization* (LMN). Finally, note that the normalisation does not have to be performed as a separate step when the UCNR method is used for testing for differential expression, because testing and normalisation are combined in the unified statistical framework.

### 3.5 Normalization using reference genes

Many normalization strategies have been described in literature. Both MOD and LMN require a large number and unbiased set of genes to be profiled. An empirical approach to select stably expressed reference genes for normalization purposes has previously been described using a gene-stability measure based on the mean pairwise variation between a given candidate reference gene and other tested candidate reference genes [11]. In the field of microRNA gene expression, however, only a few candidate reference microRNAs are reported and small non-coding RNAs are often used instead. This approach assumes however that none of these small RNAs are differentially regulated in the experiment. The selection of reference microRNAs is thus rather empirical. Large scale microRNA expression profiling studies global mean expression value normalization is characterized by a high expression stability and thus results in an adequate removal of technical variability [4]. This normalization procedure avoids the necessity of identifying good reference RNAs. For small datasets, the procedure using reference genes [11] is often the default procedure. In [4], a strategy to identify stably expressed microRNAs is presented.

The UCNR Eq (2) can be easily adapted to perform normalization using multiple reference genes. First, we introduce an indicator *R*_*i*_ for the reference genes:

$$R_{i}=\{\begin{array}{c} 0:\;\text{gene}\;i\;\text{is}\;\text{of}\;\text{interest}\\ 1:\;\text{gene}\;i\;\text{is}\;\text{a}\;\text{reference}\;\text{gene}. \end{array}$$

Let C* again refer to the latent *Cq*. Then, the UCNR with normalization based on reference makes use of

$$\begin{array}{c} C_{ijk}^{*}=\mu +\alpha_{i}+\beta_{j}+R_{i}\zeta_{j}+\left(1-R_{i}\right){\left(\alpha \gamma \right)}_{ik}+\varepsilon_{ij}, \end{array}$$ (3)

where the interpretations of the parameters *μ*, *α*_*i*_ and (*αγ*)_*ik*_ and the error term *ε*_*ij*_ remain as for model (2), and *μ* + *β*_*j*_ + *ζ*_*j*_ now represents the normalization factor. The parameter of interest, differential expression *δ*_*i*_ of gene *i*, is again a contrast of the parameters and it is estimated simultaneously with normalization. As before, generalized Wald tests can be used for hypothesis testing (further details in S1 Appendix).

### 3.6 Case study I: Upregulation in the miR-17-92 cluster in *MYCN* amplified cancer cells

The use of model (2) is illustrated by analyzing the NB dataset [4] to detect up- and downregulated microRNAs between MNA and MNSC tumor samples. The LOD was set to 35, as values above this threshold were considered to be noise [12]. This corresponds with 32.5% censored observations. The parameters of the UCNR model are again estimated using maximum likelihood estimation which allows for potential heteroskedasticity between the microRNAs.

*MYCN* amplification is the most prominent genetic alteration in neuroblastoma. Here we focus on the *miR-17-92* cluster, which is known to be upregulated in the *MYCN* amplified tumors [13]. The miR-17-92 cluster is among the first microRNAs recognized as key components of a molecular network that impacts tumorigenesis and tumor maintenance [14]. Table 1 lists the results of the differential expression analysis for the miR-17-92 cluster. All microRNAs from the cluster were found to be significantly upregulated in the MNA tumor samples (at the false discovery rate level of 5%). These findings are similar as described in [4] and [6], except for miR-17-3p which is now also found as differentially expressed. This makes sense because the entire cluster is simultaneously transcribed.

**Table 1:** Differential expression analysis with UCNR in the *miR-17-92 cluster*. The 8 microRNAs are upregulated in the MNA tumor samples. For each individual microRNA in the cluster, estimates ${\hat{\delta }}_{i}$ of the log_2_ fold change (MNSC—MNA), adjusted *p*-values (correcting for multiple testing according to [15]) and 5% false discovery rate-adjusted confidence intervals [16] for the average fold change are given.

	${\hat{\delta }}_{i}$	adj. *p*-value (BH)	5% FDR-adjusted CI for the avg FC
hsa-mir-17-3p	0.59	3.58 × 10^−5^	[1.21; 1.86]
hsa-mir-17-5p	1.04	1.16 × 10^−6^	[1.49; 2.85]
hsa-mir-18a	1.16	1.89 × 10^−6^	[1.55; 3.24]
hsa-mir-18a*	1.11	3.75 × 10^−9^	[1.63; 2.88]
hsa-mir-19a	1.33	4.34 × 10^−11^	[1.85; 3.44]
hsa-mir-19b	1.06	7.95 × 10^−8^	[1.55; 2.82]
hsa-mir-20a	1.35	3.73 × 10^−11^	[1.87; 3.47]
hsa-mir-92	1.83	2.12 × 10^−20^	[2.61; 4.84]

### 3.7 Case study II: Differential gene expression analysis of a multigene-expression signature for patients with neuroblastoma

In a second case study, we demonstrate model (3) for a multigene-expression signature that serves as a risk predictor for patients with neuroblastoma [5]. The signature supports 59 genes that were carefully selected using an innovative data-mining strategy. The prediction model was built using 30 training samples, randomly selected from a cohort of 343 neuroblastoma patients from the SIOPEN study. We perform a differential expression analysis on 58 prognostic genes on the 30 training samples (15 deceased high-risk (HR) and 15 low-risk patients (LR) with a long progression-free survival time). Furthermore, 5 reference genes (*AluSq*, *HMBS*, *HPRT1*, *SDHA*, and *UBC*) are included for normalization.

The LOD is set at 39. This choice is based on the application of a data-driven LOD selection criterion to the COG data (see S1 Appendix). Since the COG data and the SIOPEN data were generated on the same platform, an appropriate LOD for the former is expected to be good for the latter too. Moreover, by using an independent dataset for the selection of the LOD, the statistical inference procedures described earlier in the paper (e.g. hypothesis testing) remain valid. The selection of LOD = 39 was also confirmed as follows. With the SIOPEN data, a 95% confidence interval for the average *Cq* value for the detection of 1 molecule for an individual gene, based on the *y*-intercepts of a 5-point 10-fold serial dilution standard curve, is given by [37.90; 38.32] (qbase^PLUS^ version 2.4). This gives a biological justification for the choice of 39. With a LOD of 39, 2.5% of the observations are censored. Differential expression analysis between HR and LR was performed by the UCNR method.

**Fig 5:**
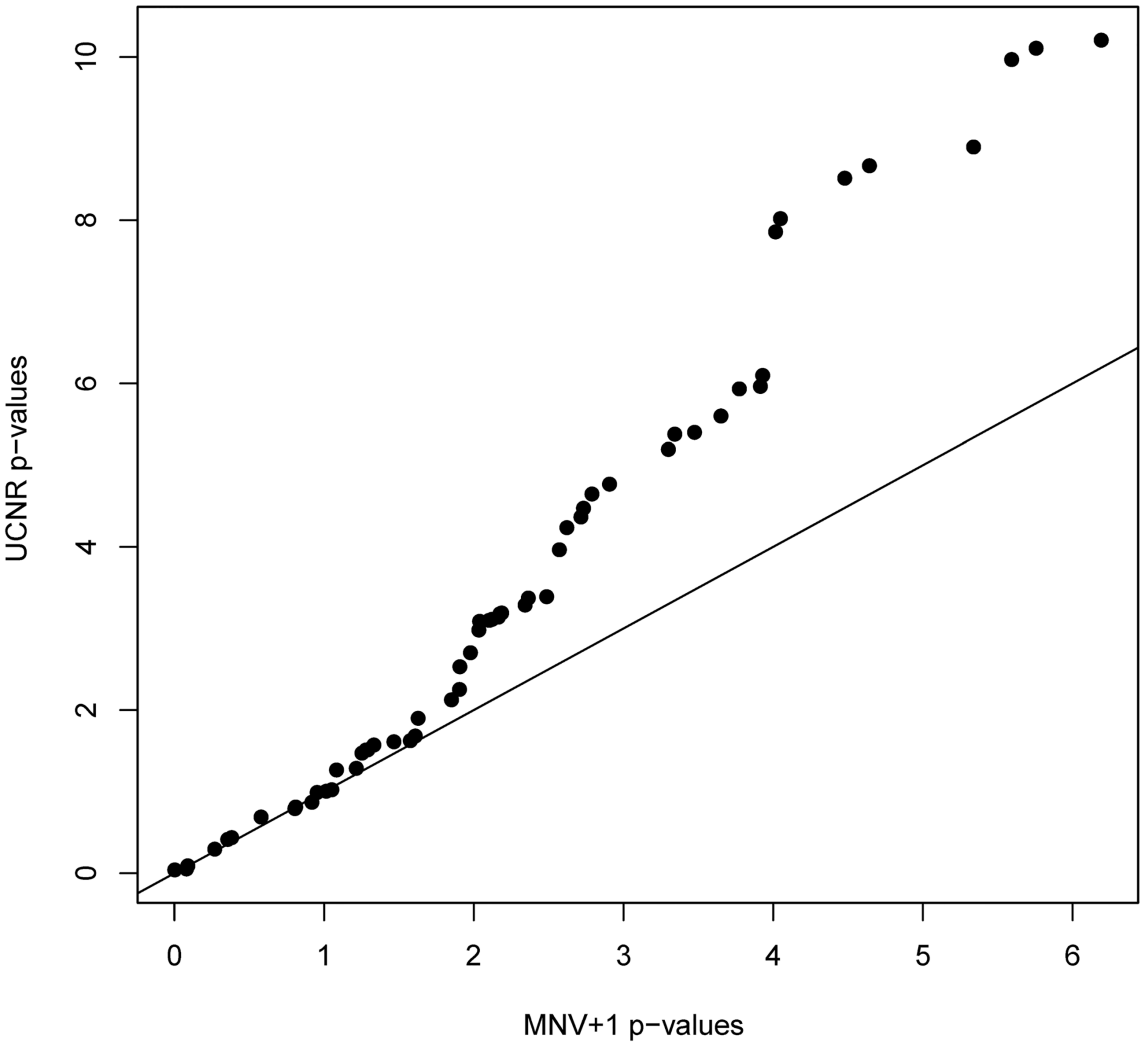
Q-Q plot of the −log_10_ *p*-values for the UCNR model (3) versus MNV+1. The *p*-values result from the differential gene expression analysis of the multigene-expression signature for patients with neuroblastoma. The solid line represents the bisector. The UCNR method typically has larger −log_10_ *p*-values than the MVN+1 method, resulting in a higher sensitivity.

The UCNR detects 43 out of the 58 genes as differentially expressed (5% false discovery rate) between the HR and LR group. A full listing of the analysis results is available as supplementary material (S1 Table). The analysis was also performed with the classical MNV+1 and LOD on the normalized data, using reference gene normalization. Both analyses detect 38 differentially expressed genes. Fig 5 displays a Q-Q plot of the -log_10_ transformed *p*-values from the UCNR model (3) versus those from the classical MNV+1. The figure illustrates that the UCNR method has very often larger -log_10*p*_ values (i.e. smaller *p* values). Since UCNR correctly controls for the type I error, the method thus guarantees a higher sensitivity. Table 2 compares the number of called significant and non-significant genes for both analyses (UCNR Eq (3) and MNV+1). UCNR Eq (3) detects 5 (7-2) extra differential expressed genes.

**Table 2:** Differential gene expression analysis of the multigene-expression signature for patients with neuroblastoma. Comparison of the significant (S) and non-significant (NS) differential expressed genes (5% false discovery rate) by UCNR model (3) versus MNV+1. UCNR detects 5 extra differential expressed genes.

		MNV+1	
		NS	S
UCNR	NS	13	2
	S	7	36

## 4 Discussion

We present a unified censored normal regression (UCNR) model for assessing differential expression in qPCR experiments. The method acts on the raw *Cq*-values. It performs normalization and differential expression analysis simultaneously while providing a statistical rigorous way for handling undetermined *Cq* values (UV). Generalized Wald tests are used for assessing differential expression and the model parameters of interest have an interpretation in terms of log_2_ fold changes.

Ignoring censoring will generally lead to inconsistent estimators [8]. Figs 1 to 4 illustrate the influence of censoring on the differential expression estimator, the normalization factor estimator and the hypothesis tests.

Sequential analyses are sensitive to censored observations as suggested by the fluctuations in the estimates of differential expression in Fig 1. The impact of ignoring censoring on two representative differentially expressed microRNAs (true |*δ*_*i*_| = 2) is illustrated in Figs 2 and 3: the *p*-values and estimates of the log_2_ fold change remain more stable with increasing degree of censoring with the UCNR method than with the other analysis methods. Figs 2 and 3 show the results of a microRNA with heavy censoring in the sense that these particular microRNAs are removed from the study after about 300-350 steps of reducing the LOD. Note that all methods result in biased log_2_ fold change estimates and low power to detect differential expression when censoring reaches levels so that few samples with concentrations above the LOD remain. For microRNAs containing less censored observations, or when the microRNA is not differentially expressed, UCNR still outperforms the other methods, but the differences between the methods are smaller (S4 and S5 Figs).

The proportion of censored observations in a microRNA before a type II error (false negative) or type I error (false positive) occurred, is used to measure the robustness of the test. For individual microRNAs, the proportions resulting from our model are never smaller and mostly larger than for the classical analyses, indicating its robustness (less sensitive to undetermined values).

The UCNR method extends the common normalization strategies, such as modified global mean normalization and the usage of reference genes or genes resembling the mean. Classical approaches first normalize the data to remove technical variability and statistical analyses are conducted on the normalized data. As a result, the standard errors of the fold change estimates are incorrectly estimated. Our method accounts for estimating the normalization factor. When normalizing using reference genes, the unified model uses the reference genes throughout the full analysis, resulting in a larger sample size and more degrees of freedom. This also affects the *p*-values. Classical sequential analyses are thus found to be more conservative than the unified method with reference genes. Note that the unified method also correctly controls the type I error rate. Hence, the power gain does not come at the expense of false positives.

Large scale experiments with many targets being measured are less vulnerable for incorrect standard errors estimates, even when a sequential approach is applied. From a theoretical perspective, correct standard error estimates and *p*-values can be obtained from a sequential procedure by applying an adjustment factor and corrected degrees of freedom. Note, however, that after these adjustments, the sequential approaches will still suffer from lower accuracy and precision in the presence of UV.

The UCNR method is successfully applied on a large scale neuroblastoma study to detect up and down regulated microRNAs between *MYCN* amplified (MNA) and *MYCN* single copy (MNSC) tumor samples. We compared the results for the MNA upregulated miR-17-92 cluster with the results obtained by the sequential analyses using multiple *t*-tests after MOD normalization and imputation of UV according to LOD and MNV+1 (S2 and S3 Tables). The findings are similar, except for miR-17-3p, which is not detected as a differentially expressed microRNA with the classical approaches. The estimates of the log_2_ fold change differences obtained with UCNR are considerably larger. UCNR employs the information contained in the censored observations throughout the full analysis, resulting in more robust and efficient estimators in the presence of UV.

The method is also applied on a mRNA neuroblastoma dataset for detecting differentially expressed genes within a 58 gene-expression signature. Reference gene normalization was used. Since the signature is validated as an accurate risk predictor for patients with neuroblastoma, it is expected that most genes are differentially expressed between the high-risk and the low-risk group. UCNR illustrates its power by detecting more differential expressed genes than with the classical analyses.

The LOD censoring threshold plays a non-ignorable role in a censored regression context. In the SIOPEN case study, an optimal LOD was selected through the evaluation of a data-driven loglikelihood-based criterion on two independent datasets that were profiled on a similar platform as the SIOPEN data. Both analyses rendered an optimal LOD which could be biologically validated.

Since the UCNR model relies on a normal distributed process, the method is thus only applicable when the assumption of normality is not violated. The same holds for analyses with multiple *t*-tests. However, in the absence of the normality assumption the properties of the estimator still hold asymptotically (i.e. for a large number of observations). If the UV results from technical error such as failure of amplification rather than a concentration below the LOD, an additional assumption of this technical failure being random (i.e. the failure of quantification is not related to the concentration, the particular target, … being assessed) is needed.

Since the UCNR model is basically a linear regression model, it can also be adapted to more complex study designs (e.g. *k*-group designs) or can be extended by including one or more confounder variables. The method guarantees to correctly account for the normalization which is simultaneously performed with the estimation.

The R code and data used to conduct the simulation and case studies are available in a GitHub repository accessible at https://github.com/CenterForStatistics-UGent/UCNR. The case studies have been documented so that they can be adapted to analyse the users’ own data.

## 5 Conclusion

We proposed a unified censored normal regression (UCNR) model for analyzing differential expression in qPCR experiments. The model acts on the raw *Cq*-values and accounts for undetermined values (UV) without requiring ad hoc imputation algorithms. The model integrates the normalization procedure within the statistical analysis. We showed that the estimator and hypothesis tests are robust in the presence of UV and that our method outperforms popular imputation methods in terms of accuracy and precision.

## Supporting information

S1 Fig Bias (left) and root mean squared errors (RMSE) (right) of the differential expression estimates of 100 microRNAs, as a function of the number of censored Cq values. At the bottom the grey circles indicate the removal of a complete miRNA (as a consequence of censoring). The numbers on top of some of the grey circles represent the number of remaining miRNA in the study. Estimators are obtained by UCNR (green solid line), multiple *t*-tests with MOD normalization and LOD imputation of the UV (red dashed line), multiple *t*-tests with MOD normalization and MNV+1 imputation (blue dotted line) and multiple *t*-tests with MOD normalization and KNN imputation (black dotted-dashed line). A bias closer to zero suggest more accurate estimates. A small RMSE indicate a high precision of the estimator. The sharp jumps in the curves happen when a complete miRNA gets censored, which heavily affects the normalisation constants.
(PDF)

S2 Fig Bias (left) and root mean squared errors (RMSE) (right) of the differential expression estimates of 200 microRNAs, as a function of the number of censored Cq values. At the bottom the grey circles indicate the removal of a complete miRNA (as a consequence of censoring). The numbers on top of some of the grey circles represent the number of remaining miRNA in the study. Estimators are obtained by UCNR (green solid line), multiple *t*-tests with MOD normalization and LOD imputation of the UV (red dashed line), multiple *t*-tests with MOD normalization and MNV+1 imputation (blue dotted line) and multiple *t*-tests with MOD normalization and KNN imputation (black dotted-dashed line). A bias closer to zero suggest more accurate estimates. A small RMSE indicate a high precision of the estimator. The sharp jumps in the curves happen when a complete miRNA gets censored, which heavily affects the normalisation constants.
(PDF)

S3 Fig Two differentially expressed microRNAs (true *δ*_*i*_ = 2 (up) and *δ*_*i*_ = −2 (down)) tracked during the simulation study. Plot of −log_10_(*p*)-values for the hypothesis tests: UCNR (green solid line) and Wilcoxon rank sum after MNV+1(blue dotted line), LOD (red dashed line) and KNN (black dotted-dashed line normalisation.
(PDF)

S4 Fig The graphs illustrate the differences between the methods when applied to differentially expressed microRNAs that show only minor censoring. Two differentially expressed microRNAs (true |*δ*_*i*_| = 2) are tracked during the simulation study. (a) Estimates of differential expression by UCNR (green solid line), multiple *t*-tests with MOD normalization and LOD imputation (red dashed line), MNV+1 imputation (blue dotted line) and KNN imputation (black dotted-dashed line). Censoring an observation at some point for this particular microRNA is marked by a black circle (MNA group) or a grey square (MNSC group) on the horizontal axis. (b) Plot of −log_10_ *p*-values for the hypothesis test (*H*_0_: *δ*_*i*_ = 0;*H*_1_: *δ*_*i*_ ≠ 0). (c) Box plot of differential expression estimates.
(PDF)

S5 Fig The graphs illustrate the differences between the methods when applied to non-differentially expressed microRNAs (true *δ*_*i*_ = 0). Two non-differentially expressed microRNAs are tracked during the simulation study. (a) Estimates of differential expression by UCNR (green solid line), multiple *t*-tests with MOD normalization and LOD imputation (red dashed line), MNV+1 imputation (blue dotted line) and KNN imputation (black dotted-dashed line). Censoring an observation at some point for this particular microRNA is marked by a black circle (MNA group) or a grey square (MNSC group) on the horizontal axis. (b) Plot of −log_10_ *p*-values for the hypothesis test (*H*_0_: *δ*_*i*_ = 0;*H*_1_: *δ*_*i*_ ≠ 0). (c) Box plot of differential expression estimates.
(PDF)

S1 Table Results for SIOPEN data (UCNR). The table shows parameter estimates and their standard errors (SE) for each of the 58 microRNAs, as well as the two-sided *p*-values and adjusted *p*-values (using the Benjamini and Hochberg procedure) for testing for no differential expression. 43 out of the 58 microRNAs are differentially expressed at the 5% false discovery rate.
(PDF)

S2 TableResults for the miR-17-92 cluster, using *t*-tests after MOD normalization and LOD imputation of UV.The table shows the estimated log_2_ fold change (${\hat{\delta }}_{i}$), the *p*-value and the adjusted *p*-value (Benjamini and Hochberg correction).(PDF)

S3 TableResults for the miR-17-92 cluster, using *t*-tests after MOD normalization and MNV+1 imputation of UV.The table shows the estimated log_2_ fold change (${\hat{\delta }}_{i}$), the *p*-value and the adjusted *p*-value (Benjamini and Hochberg correction).(PDF)

S1 Appendix Derivations of differential expression in the unified censored regression model and the selection of an optimal LOD. (PDF)
