# Supplementary material for: A unified censored normal regression model for qPCR differential gene expression analysis
Source: PLoS One. 2017 Aug 17;12(8):e0182832. doi: 10.1371/journal.pone.0182832 (PMC5560691; doi:10.1371/journal.pone.0182832)
Supplement: S1 Fig — At the bottom the grey circles indicate the removal of a complete miRNA (as a consequence of censoring). The numbers on top of some of the grey circles represent the number of remaining miRNA in the study. Estimators are obtained by UCNR (green solid line), multiple t-tests with MOD normalization and LOD imputation of the UV (red dashed line), multiple t-tests with MOD normalization and MNV+1 imputation (blue dotted line) and multiple t-tests with MOD normalization and KNN imputation (black dotted-dashed line). A bias closer to zero suggest more accurate estimates. A small RMSE indicate a high precision of the estimator. The sharp jumps in the curves happen when a complete miRNA gets censored, which heavily affects the normalisation constants. (PDF) [file pone.0182832.s001.pdf]

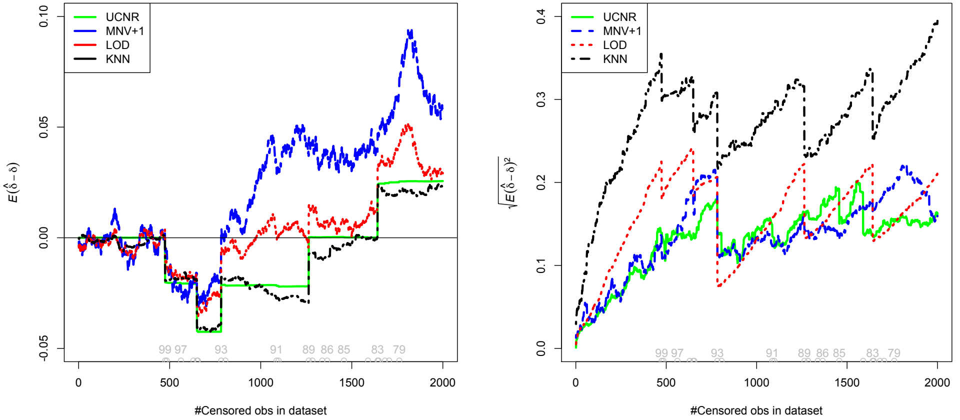

S1 Figure: Bias (left) and root mean squared errors (RMSE) (right) of the differential expression estimates of 100 microRNAs, as a function of the number of censored *Cq* values. At the bottom the grey circles indicate the removal of a complete miRNA (as a consequence of censoring). The numbers on top of some of the grey circles represent the number of remaining miRNA in the study. Estimators are obtained by UCNR (green solid line), multiple *t*-tests with MOD normalization and LOD imputation of the UV (red dashed line), multiple *t*-tests with MOD normalization and MNV+1 imputation (blue dotted line) and multiple *t*-tests with MOD normalization and KNN imputation (black dotted-dashed line). A bias closer to zero suggest more accurate estimates. A small RMSE indicate a high precision of the estimator. The sharp jumps in the curves happen when a complete miRNA gets censored, which heavily affects the normalisation constants.
