# Supplementary material for: A unified censored normal regression model for qPCR differential gene expression analysis
Source: PLoS One. 2017 Aug 17;12(8):e0182832. doi: 10.1371/journal.pone.0182832 (PMC5560691; doi:10.1371/journal.pone.0182832)
Supplement: S3 Fig — Plot of −log10(p)-values for the hypothesis tests: UCNR (green solid line) and Wilcoxon rank sum after MNV+1(blue dotted line), LOD (red dashed line) and KNN (black dotted-dashed line normalisation. (PDF) [file pone.0182832.s003.pdf]

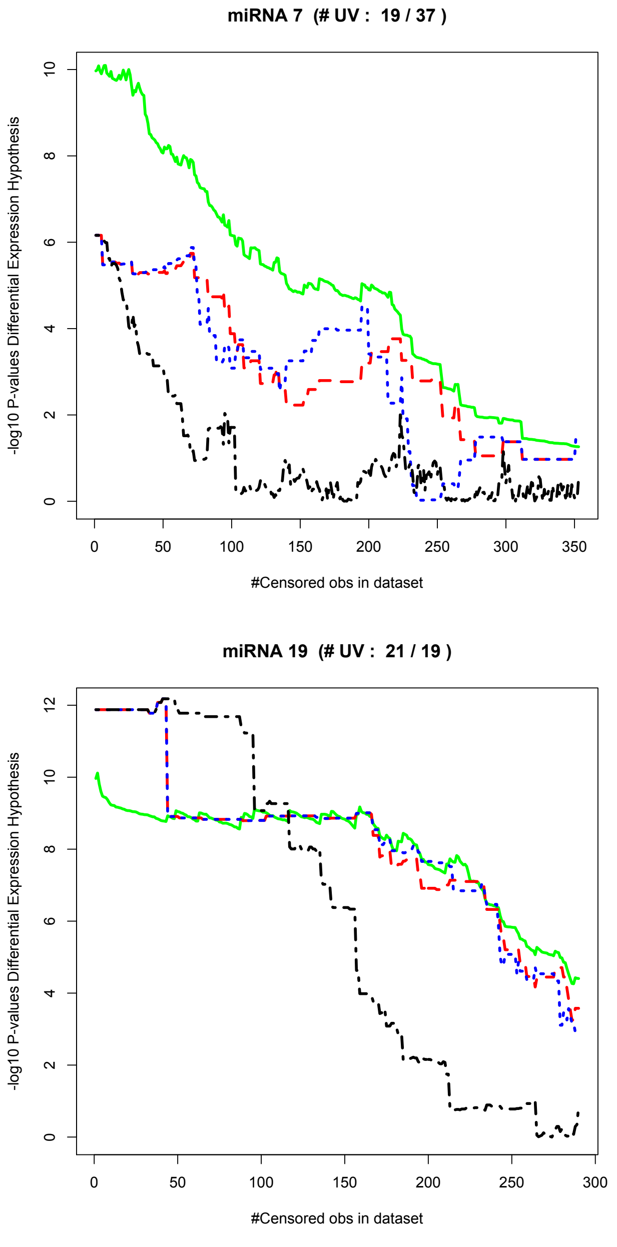

S3 Figure: Two differentially expressed microRNAs (true$\delta_i = 2$ (up) and$\delta_i = -2$ (down)) tracked during the simulation study. Plot of$-\log_{10}(p)$-values for the hypothesis tests: UCNR (green solid line) and Wilcoxon rank sum after MNV+1(blue dotted line), LOD (red dashed line) and KNN (black dotted-dashed line normalisation.
