# Supplementary material for: A unified censored normal regression model for qPCR differential gene expression analysis
Source: PLoS One. 2017 Aug 17;12(8):e0182832. doi: 10.1371/journal.pone.0182832 (PMC5560691; doi:10.1371/journal.pone.0182832)
Supplement: S5 Fig — Two non-differentially expressed microRNAs are tracked during the simulation study. (a) Estimates of differential expression by UCNR (green solid line), multiple t-tests with MOD normalization and LOD imputation (red dashed line), MNV+1 imputation (blue dotted line) and KNN imputation (black dotted-dashed line). Censoring an observation at some point for this particular microRNA is marked by a black circle (MNA group) or a grey square (MNSC group) on the horizontal axis. (b) Plot of −log10 p-values for the hypothesis test (H0: δi = 0;H1: δi ≠ 0). (c) Box plot of differential expression estimates. (PDF) [file pone.0182832.s005.pdf]

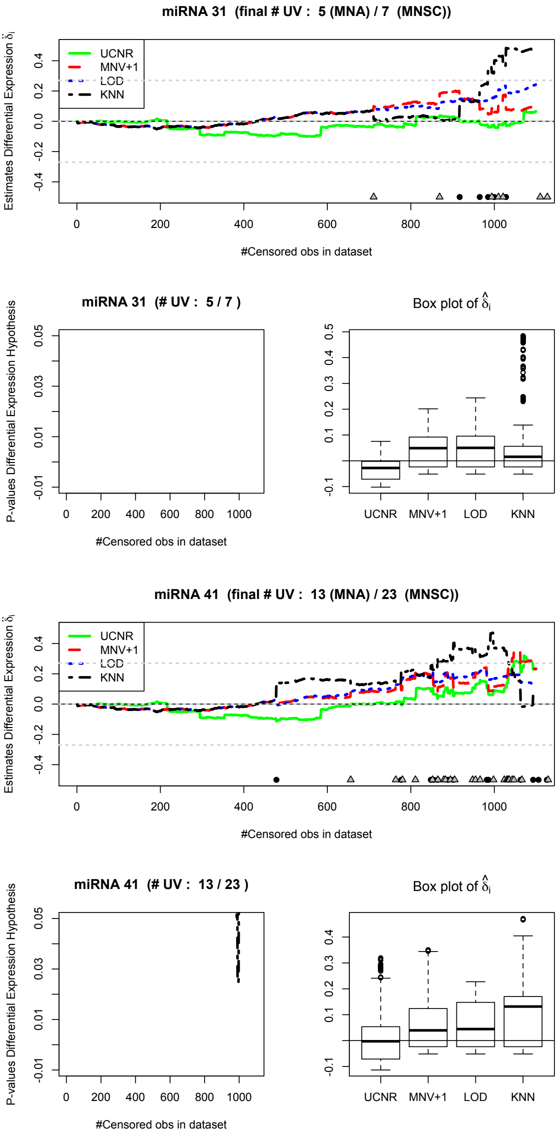

S5 Figure: The graphs illustrate the differences between the methods when applied to non-differentially expressed microRNAs (true$\delta_i = 0$). Two non-differentially expressed microRNAs are tracked during the simulation study. (a) Estimates of differential expression by UCNR (green solid line), multiple t-tests with MOD normalization and LOD imputation (red dashed line), MNV+1 imputation (blue dotted line) and KNN imputation (black dotted-dashed line). Censoring an observation at some point for this particular microRNA is marked by a black circle (MNA group) or a grey square (MNSC group) on the horizontal axis. (b) Plot of $-\log_{10} p$-values for the hypothesis test $(H_0 : \delta_i = 0; H_1 : \delta_i \neq 0)$. (c) Box plot of differential expression estimates.
