# Supplementary material for: A unified censored normal regression model for qPCR differential gene expression analysis
Source: PLoS One. 2017 Aug 17;12(8):e0182832. doi: 10.1371/journal.pone.0182832 (PMC5560691; doi:10.1371/journal.pone.0182832)
Supplement: S1 Table — The table shows parameter estimates and their standard errors (SE) for each of the 58 microRNAs, as well as the two-sided p-values and adjusted p-values (using the Benjamini and Hochberg procedure) for testing for no differential expression. 43 out of the 58 microRNAs are differentially expressed at the 5% false discovery rate. (PDF) [file pone.0182832.s006.pdf]

Gene	Estimate	SE	p-value	adjusted p-value (BH)
AHCY	-1.46	0.25	0.00	0.00
AKR1C1	3.63	0.56	0.00	0.00
ARHGEF7	0.77	0.55	0.16	0.18
BIRC5	-2.28	0.56	0.00	0.00
CADM1	0.39	0.43	0.36	0.40
CAMTA1	2.24	0.37	0.00	0.00
CAMTA2	1.15	0.51	0.02	0.04
CD44	1.75	0.57	0.00	0.00
CDCA5	-1.21	0.95	0.20	0.23
CDKN3	-1.59	0.46	0.00	0.00
CHD5	8.01	1.32	0.00	0.00
CLSTN1	2.56	0.92	0.01	0.01
DDC	3.46	0.86	0.00	0.00
DPYSL3	1.58	0.28	0.00	0.00
ECEL1	3.83	0.79	0.00	0.00
ELAVL4	1.12	0.48	0.02	0.03
EPB41L3	2.98	0.66	0.00	0.00
EPHA5	5.14	1.57	0.00	0.00
EPN2	2.83	0.67	0.00	0.00
FYN	0.88	0.26	0.00	0.00
GNB1	0.56	0.26	0.03	0.05
HIVEP2	2.27	0.59	0.00	0.00
INPP1	1.23	0.57	0.03	0.04
MAP2K4	0.57	0.34	0.09	0.12
MAP7	2.72	0.80	0.00	0.00
MAPT	2.25	0.49	0.00	0.00
MCM2	-1.68	0.48	0.00	0.00
MRPL3	-0.74	0.28	0.01	0.01
MTSS1	1.03	0.41	0.01	0.02
MYCN	-2.12	0.96	0.03	0.04
NHLH2	-7.94	4.08	0.05	0.07
NME1	-1.52	0.32	0.00	0.00
NRCAM	2.23	0.38	0.00	0.00
NTRK1	6.75	1.04	0.00	0.00
ODC1	-2.18	0.33	0.00	0.00
PAICS	-0.64	0.45	0.15	0.18
PDE4DIP	1.27	0.38	0.00	0.00
PIK3R1	1.41	0.40	0.00	0.00
PLAGL1	1.59	0.96	0.10	0.12
PLAT	2.73	1.26	0.03	0.04
PMP22	2.00	0.59	0.00	0.00
PRAME	-3.47	1.02	0.00	0.00
PRDM2	0.27	0.31	0.38	0.41
PRKACB	1.09	0.48	0.02	0.04
PRKCZ	1.20	0.40	0.00	0.01
PTN	1.13	0.75	0.13	0.16
PTPRF	1.06	0.65	0.10	0.12
PTPRH	5.41	1.18	0.00	0.00
PTPRN2	2.93	0.68	0.00	0.00
QPCT	2.70	0.65	0.00	0.00
SCG2	2.65	0.78	0.00	0.00
SLC25A5	-1.52	0.31	0.00	0.00
SLC6A8	0.22	0.32	0.50	0.53
SNAPC1	-0.11	0.45	0.81	0.84
TNFRSF25	0.11	0.72	0.88	0.90
TYMS	-1.94	0.40	0.00	0.00
ULK2	0.69	0.36	0.05	0.07
WSB1	0.06	0.47	0.90	0.90

S1 Table: Results for SIOPEN data (UCNR). The table shows parameter estimates and their standard errors (SE) for each of the 58 microRNAs, as well as the two-sided p-values and adjusted p-values (using the Benjamini and Hochberg procedure) for testing for no differential expression. 43 out of the 58 microRNAs are differentially expressed at the 5% false discovery rate.
