# Supplementary material for: A unified censored normal regression model for qPCR differential gene expression analysis
Source: PLoS One. 2017 Aug 17;12(8):e0182832. doi: 10.1371/journal.pone.0182832 (PMC5560691; doi:10.1371/journal.pone.0182832)
Supplement: S2 Table — The table shows the estimated log2 fold change (δ^i), the p-value and the adjusted p-value (Benjamini and Hochberg correction). (PDF) [file pone.0182832.s007.pdf]

	$\hat{\delta}_i$	p-value	adj. p-value (BH)
hsa-mir-17-3p	0.15	0.35	0.49
hsa-mir-17-5p	0.60	0.02	0.07
hsa-mir-18a	0.72	0.01	0.05
hsa-mir-18a*	0.69	0.00	0.01
hsa-mir-19a	0.90	0.00	0.00
hsa-mir-19b	0.63	0.01	0.04
hsa-mir-20a	0.91	0.00	0.01
hsa-mir-92	1.20	0.00	0.01

S2 Table: Results for the miR-17-92 cluster, using t-tests after MOD normalization and LOD imputation of UV. The table shows the estimated $\log_2$ fold change ($\hat{\delta}_i$), the p-value and the adjusted p-value (Benjamini and Hochberg correction).
