# Supplementary material for: A unified censored normal regression model for qPCR differential gene expression analysis
Source: PLoS One. 2017 Aug 17;12(8):e0182832. doi: 10.1371/journal.pone.0182832 (PMC5560691; doi:10.1371/journal.pone.0182832)
Supplement: S3 Table — The table shows the estimated log2 fold change (δ^i), the p-value and the adjusted p-value (Benjamini and Hochberg correction). (PDF) [file pone.0182832.s008.pdf]

| | $\hat{\delta}_i$ | p-value | adj. p-value (BH) |
|---|---|---|---|
| hsa-mir-17-3p | 0.15 | 0.35 | 0.50 |
| hsa-mir-17-5p | 0.60 | 0.02 | 0.07 |
| hsa-mir-18a | 0.72 | 0.01 | 0.05 |
| hsa-mir-18a* | 0.69 | 0.00 | 0.01 |
| hsa-mir-19a | 0.90 | 0.00 | 0.00 |
| hsa-mir-19b | 0.63 | 0.01 | 0.03 |
| hsa-mir-20a | 0.91 | 0.00 | 0.00 |
| hsa-mir-92 | 1.20 | 0.00 | 0.01 |

S3 Table: Results for the miR-17-92 cluster, using $t$-tests after MOD normalization and MNV+1 imputation of UV. The table shows the estimated $\log_2$ fold change ($\hat{\delta}_i$), the $p$-value and the adjusted $p$-value (Benjamini and Hochberg correction).
