# Supplementary material for: A unified censored normal regression model for qPCR differential gene expression analysis
Source: PLoS One. 2017 Aug 17;12(8):e0182832. doi: 10.1371/journal.pone.0182832 (PMC5560691; doi:10.1371/journal.pone.0182832)
Supplement: S1 Appendix — (PDF) [file pone.0182832.s009.pdf]

Supplementary to A Unified Censored Normal Regression Model for qPCR Differential Gene Expression Analysis

S1 Appendix

Differential expression in the unified censored regression model

Differential expression in model 2 (eq. 2, main paper)

We discuss the equivalence in estimating differential expression with the UCNR model 2 (eq. 2, main paper) and with the sequential analyses with multiple t-tests, in the case of no censoring (i.e. all $C = C^* \leq$LOD). In this situation model 2 (eq. 2, main paper) also applies to the observed Cq values (C) and the maximum likelihood estimates are equivalent to the least squares estimates.

We consider two groups of subjects, K_1 ($k = 1$) and K_2 ($k = 2$) with sample sizes n_1 and n_2, respectively, with the following restrictions imposed:

$$\sum_i \alpha_i = 0; \sum_j \beta_j = 0; \sum_i (\alpha\gamma)_{i2} = 0 \text{ and } (\alpha\gamma)_{i1} = 0.$$

The differential expression parameter is then $\delta_i = (\alpha\gamma)_{i2} - (\alpha\gamma)_{i1}$, with estimate

$$\begin{aligned} \hat{\delta}_i &= (\widehat{\alpha\gamma})_{i2} - (\widehat{\alpha\gamma})_{i1} \\ &= \frac{1}{n_2} \sum_{j' \in K_2} \left(C_{ij'2} - \hat{\beta}_{j'} \right) - \frac{1}{n_1} \sum_{j \in K_1} \left(C_{ij1} - \hat{\beta}_j \right), \end{aligned} \quad (1)$$

where the estimate $\hat{\beta}_j$ coincides with the latent mean normalization factor. If no censoring is encountered, MOD and LMN are identical and point estimates from model 2 (eq. 2, main paper) and the sequential analyses are equal.

Differential expression in model 3 (eq. 3, main paper)

Model 3 (eq. 3, main paper) incorporates reference genes for normalization purposes. As in the previous appendix, we assume that no censoring occurs. The sum $\mu + \beta_j + \zeta_j$ now represents the normalization factor based on the arithmic mean (log scale) of the reference genes or genes resembling the mean.

Consider again two groups of subjects, K_1 ($k = 1$) and K_2 ($k = 2$) with sample sizes n_1 and n_2, respectively, and let $\mathcal{G}$ denote the index set of genes of interest (thus, $R_i = 0$ for $i \in \mathcal{G}$). The following restrictions are imposed:

$$\sum_i \alpha_i = 0; \sum_{i \epsilon \mathcal{G}} (\alpha\gamma)_{i2} = 0 \text{ and } (\alpha\gamma)_{i1} = 0.$$

The differential expression parameter is given by

$$\delta_i = (\alpha\gamma)_{i2} - \frac{1}{n_2} \sum_{j' \in K_2} \zeta_{j'} - \left((\alpha\gamma)_{i1} - \frac{1}{n_1} \sum_{j \in K_1} \zeta_j \right)$$

which is estimated as

$$\hat{\delta}_i = \frac{1}{n_2} \sum_{j' \in K_2} \left(C_{ij'2} - \hat{\beta}_{j'} - \hat{\zeta}_{j'} \right) - \frac{1}{n_1} \sum_{j \in K_1} \left(C_{ij1} - \hat{\beta}_j - \hat{\zeta}_j \right).$$

The sum $\hat{\mu} + \hat{\beta}_j + \hat{\zeta}_j$ is equivalent to the latent mean normalization factor according to the reference genes.

Selection of an optimal LOD

The censoring threshold (LOD) in the UCNR is assumed to be fixed before the start of the study, but a high threshold may result in the inclusion of too much noisy data, resulting in a reduction of the power of the statistical tests. We suggest to use a selection criterion to determine an optimal censoring threshold.

The loglikelihood function of the UCNR model is an appropriate goodness-of-fit measure. However, as will be shown in the next paragraph, the loglikelihood can be decomposed into two components, say l^o and l^p, and the l^p component does not suffer from the monotonicity drawback while still retaining its goodness-of-fit interpretation.

Since the LOD must apply to all targets and to all samples, the loglikelihood will be considered for a UCNR model without any group effects. The index k in model 2 (eq. 2, main paper) and model 3 (eq. 3, main paper) may thus be dropped. The first component equals

$$l^o = \sum_{<LOD} \left(\log \phi(C_{ij} | \mu_{ij}, \sigma_i^2) - \log \Phi(LOD | \mu_{ij}, \sigma_i^2) \right), \tag{2}$$

where $\mu_{ij} = \mathrm{E}\left\{C_{ij}^*\right\} = \mu + \alpha_i + \beta_j + R_i \zeta_j$ (the final term vanishes for model 2 (eq. 2, main paper)), $\phi(.)$ and $\Phi(.)$ refer to the probability density function and the cumulative distribution function of the normal distribution, respectively, and the summation $\sum_{<LOD}$ refers to a summation over all sample observations C_{ij} smaller than the LOD. The second component is given by

$$l^p = \sum_{\geq LOD} \log\left(1 - \Phi(LOD | \mu_{ij}, \sigma_i^2)\right) + \sum_{<LOD} \log \Phi(LOD | \mu_{ij}, \sigma_i^2), \tag{3}$$

where the summation $\sum_{\geq LOD}$ refers to a summation over all sample observations C_{ij} not smaller than the LOD.

The component l^o is interpreted as the loglikelihood for the truncated regression model where the UV are excluded from the sample. The component l^p is interpreted as the loglikelihood for the probit model, which is a binary model where the response is either a censored (1) or an uncensored (0) realization. The total loglikelihood of the UCNR model is then $l = l^o + l^p$.

Whereas l^o cannot decrease with decreasing LOD, l^p does not suffer from this feature. Moreover, the l^p component is useful for selecting an LOD that gives optimal separation of the censored and uncensored observations. An optimal

LOD is then determined by fitting UCNR to the data for a set of potential LODs and subsequently selecting the LOD that maximizes l^p.

In the SIOPEN study the original LOD was 45, resulting in 1.5% censored observations. We evaluated l^p on two independent datasets (SIOPEN data without the training samples and COG) for a sequence of LODs that decrease gradually from 45 to 31. A maximum of the l^p is reached for a LOD of 39 in both analyses (Figure 1 and 2), suggesting that this LOD results in an optimal trade-off between expressed and undetermined realizations of the data. Both figures illustrate a plateau phase around the optimum, suggesting that a LOD of 38 or 40 is about equally plausible. Selecting these values as optimal LOD equally results in a higher sensitivity for UCNR.

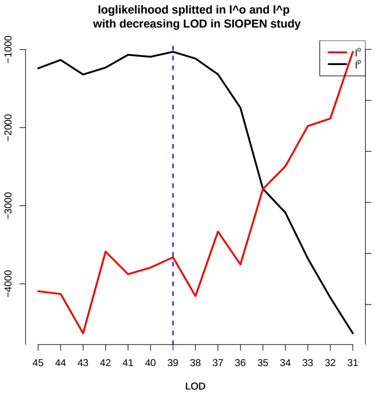

Figure 1: Plot of the loglikelihood components l^o (2) and l^p (3) for a sequence of LODs (from 45 to 31) for the SIOPEN data without the training samples. A maximum of the l^p is reached when LOD = 39. The individual contribution to the loglikelihood is read from the left axis (l^p) and the right axis (l^o).

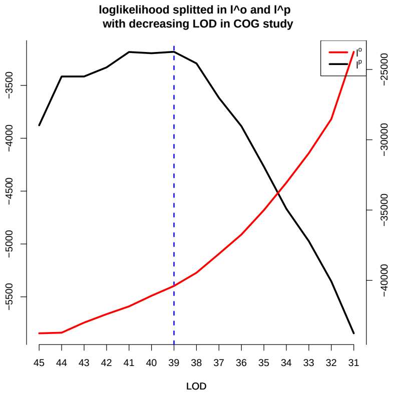

Figure 2: Plot of the loglikelihood components l^o (2) and l^p (3) for a sequence of LODs (from 45 to 31) for the COG data. A maximum of l^p is reached when LOD = 39. The individual contribution to the loglikelihood is read from the left axis (l^p) and the right axis (l^o).
